# Midwives’ cardiotocograph interpretation and documentation knowledge in a selected Gauteng hospital

**DOI:** 10.4102/hsag.v31i0.3260

**Published:** 2026-01-21

**Authors:** Sifiso Dlamini, Kagiso P. Tukisi, Roinah Ngunyulu

**Affiliations:** 1 Department of Advanced Nursing Science, Faculty of Health Sciences, University of Venda, Thohoyandou, South Africa; 2 Department of Nursing, Faculty of Health Sciences, University of Johannesburg, Johannesburg, South Africa

**Keywords:** cardiotocograph, interpretation, knowledge, documentation, midwives, Gauteng

## Abstract

**Background:**

Cardiotocography (CTG) is a globally used intrapartum monitoring tool to assess the foetal heart rate and the related responses to the physiology of the uterine action. The intrapartum foetal monitoring is the primary responsibility of the midwives caring for the woman in labour. Midwives’ knowledge and understanding of CTG traces and interpretation ensure timely responses to pathological findings, thus ensuring positive foetal and neonatal outcomes.

**Aim:**

The study aimed to assess the midwives’ knowledge of cardiotocograph interpretation and documentation in a selected hospital in Gauteng.

**Setting:**

The data were collected in a selected secondary hospital, a referral hospital for the midwife-led obstetric units and district hospitals in the Gauteng province.

**Methods:**

The study employed a quantitative retrospective research design, utilising a checklist to analyse patients’ past medical records – this retrospective analysis aimed to evaluate midwives’ knowledge in interpreting CTG.

**Results:**

A review of 336 CTG case records analysed five criteria: baseline heart rate, variability, decelerations, accelerations, and overall trace assessment. Significant discrepancies in record-keeping were found between midwives and researchers. Researchers maintained complete records and interpretations for all criteria, whereas midwives exhibited considerable gaps, ranging from 31% to 100% across the CTG criteria.

**Conclusion:**

Analysis indicates a substantial gap in CTG documentation by midwives compared to researchers.

**Contribution:**

Midwives exhibiting significant data omissions that may affect intrapartum care quality and clinical decisions.

## Introduction

Cardiotocography (CTG) is an electronic monitoring technique used to simultaneously record the foetal heart and the intensity and frequency of the uterine contractions during labour (Sellers, Dippenaar & Da Serra 2018). The physiology of labour and the related uterine contractions include the partial closure of the maternal sinuses responsible for foetal oxygenation, thus subjecting the foetus to mild hypoxia (Rosen & Yogev 2023). The mild hypoxia secondary to the physiology of labour may be in high-risk pregnancies, which heralds a need for continuous CTG monitoring for early diagnosis of hypoxia and the related foetal responses signifying compensatory mechanisms (Sellers et al. 2018).

According to the National Institute for Health and Care Excellence guidelines, the CTG assesses the foetal heart rate using the following parameters: baseline, variability, and accelerations (Rosen & Yogev 2023). In addition, the frequency and intensity of the uterine contractions and foetal response to such contractions, termed decelerations, are also recorded (David & Spencer 2022). Consequently, the presence and use of CTG enable the healthcare professional to establish a relationship between the foetal heart rate and the uterine contractions to diagnose foetal wellbeing, hypotonic, and hypertonic uterine contractions (Rosen & Yogev 2023). The use of the CTG increases the likelihood of predicting the abnormal neonatal outcomes by close to 87%, making CTG an essential diagnostic tool during labour (Das, Majumdar & Dey 2023).

Whilst the CTG is a good predictor of possible abnormal outcomes, the literature suggests a 60% chance of a false-positive rate of CTG, which renders the CTG a poor predictor of the actual foetal distress (Das et al. 2023). The false positive is subject to healthcare professionals’ accurate interpretation of the CTG, which may vary depending on an individual’s assessment (Daydulo et al. 2022).

To mitigate the inconsistencies in the interpretation of the CTG tracings, the guidelines recommend meticulous documentation of all the relevant clinical findings to provide a clinical context from which the CTG trace may be interpreted, such as existing medical disorders (Rishard et al. 2025). Significant changes in foetal heart rate patterns, including the exclusion of tachycardia and bradycardia from baseline readings, along with poor beat-to-beat variability, can provide critical insights into the foetal condition (Das et al. 2023; Rishard et al. 2025). Additionally, assessing uterine activity, such as hypotonic or hypertonic contractions, may assist in identifying early and late decelerations, further supporting the diagnosis. Therefore, the components of the CTG cannot be recorded and interpreted in isolation, making CTG interpretation complex (Chandraharan et al. 2025).

Cardiotocography trace forms part of the maternal and neonatal care clinical records (Das et al. 2023). Consequently, its recording and interpretation must be aligned to the scope of practice and conditions for midwives’ practice in South African Nursing Council (SANC) regulations (R.2488) (SANC 2022). The existing literature proves that there are inconsistencies in clinical recording, which resulted in incomplete records inadvertently compromising the interpretation of clinical data, including the CTG (Das et al. 2023). The incomplete records resulted in misinterpretation of the CTG, which could misdiagnose the actual and potential problems. Consequently, inappropriate responses to the immediate maternal and foetal needs may result, thus giving rise to deleterious outcomes (Nakao et al. 2020).

The intrapartum care guidelines discourage the use of CTG on low-risk pregnant women, requiring the referral of high-risk pregnant women to the hospital for intrapartum care (The South African Department of Health’s 2024 “National Integrated Maternal and Perinatal Care Guidelines” (5th edition)). The hospital labour wards are responsible for managing high-risk pregnancies and labour, which require the continuous monitoring of the foetal heart’s wellbeing using the CTG (DoH 2024). Consequently, the use of CTG is an integral part of high-risk intrapartum care to detect early the possibility of intrauterine hypoxia (Rosen & Yogev 2023). The intrauterine hypoxia may contribute to the hypoxic ischaemic encephalopathy and cerebral palsy, which account for 50% of the medical litigation cases (Nakao et al. 2020). Consequently, the CTG trace as a clinical record is proof in such cases. Therefore, the study sought to assess the midwives’ knowledge of cardiotocograph interpretation and documentation in the selected hospital in Gauteng.

## Research methods and design

### Research design

The study employed a quantitative retrospective research design, utilising a checklist to analyse patients’ past medical records – this retrospective analysis aimed to evaluate midwives’ knowledge in interpreting CTG.

### Setting

The study was conducted in a secondary hospital located in the Gauteng province. The selected hospital is a referral hospital for the primary healthcare clinics, community healthcare centres, and district hospitals. Consequently, the selected hospital provides intrapartum care services to high-risk pregnant women who require close foetal monitoring using CTG.

### Study population

A total of 5808 files from the selected period were accessed from the hospital’s DoH (2024) database.

### Sampling technique and sample size

A randomised sampling technique was employed to ensure this study’s findings are statistically reliable and generalisable. This method ensures that each individual in the target population has an equal chance of being selected, thereby reducing selection bias and enhancing the sample’s representativeness.

Based on standard statistical calculations and assuming a population proportion of 0.5 (which provides the most conservative estimate), a sample size of *366 participants* was determined to be sufficient. This sample size allows the study to achieve a *95% confidence level* with a *5% margin of error*, meaning the results can be interpreted with high precision and minimal risk of error. The retrieved files were organised into a month-to-month table, and data from each month were entered into an Excel spreadsheet for random file selection (Table 1).

**TABLE 1:** Selected files for analysis date date of delivery.

Date	Frequency	%	Valid %	Cumulative %
01/2024	30	8.2	8.2	8.2
02/2024	40	10.9	10.9	19.1
03/2023	28	7.7	7.7	26.8
04/2023	30	8.2	8.2	35.0
05/2023	29	7.9	7.9	42.9
06/2023	29	7.9	7.9	50.8
07/2023	30	8.2	8.2	59.0
08/2023	30	8.2	8.2	67.2
09/2023	30	8.2	8.2	75.4
10/2023	30	8.2	8.2	83.6
11/2023	30	8.2	8.2	91.8
12/2023	30	8.2	8.2	100.0

**Total**	**366**	**100.0**	**100.0**	-

*Source*: Dlamini, S., 2025, ‘Determining midwives’ knowledge of cardiotocography interpretation in selected public hospital in Gauteng: A retrospective study’, Master’s thesis, University of Johannesburg, University of Johannesburg Institutional Repository, viewed n.d., from https://hdl.handle.net/10210/516532

The archives department staff then extracted the files, and each selected file was analysed using the inclusion and motivation table. A total of366 files were selected using randomised sampling using the inclusion criteria detailed in Table 2.

**TABLE 2:** Inclusion criteria.

Inclusion criteria	Motivation
High-risk labour	CTG is conducted on high-risk women according to the maternal guidelines.
Eligible records of admission	Records should follow the documentation principles of legibility and completeness. Concise, legal prudence, and accepted terminology.
CTG strip present	The strip should be present in the file to interpret the CTG.
Notes from the midwives or midwifery specialist	The midwives are the element of the study as the primary variable to keep records of the file.
Files from women who delivered in the period of 01/2022–12/2022	The midwives focused on are presumed to be present, and the recommendations will be relevant to their records.

*Source*: Dlamini, S., 2025, ‘Determining midwives’ knowledge of cardiotocography interpretation in selected public hospital in Gauteng: A retrospective study’, Master’s thesis, University of Johannesburg, University of Johannesburg Institutional Repository, viewed n.d., from https://hdl.handle.net/10210/516532

The sample met the inclusion criteria (Table 1): high-risk labour, eligible records of admission, presence of CTG strips, notes from midwives or advanced midwives, and files from women who delivered between February 2023 and March 2024.

### Pilot study

A pilot study was conducted to assess a questionnaire’s clarity and data-soliciting capability. In consultation with a statistician, a sample of files from the post-natal ward, representing 10% of the study’s sample size (40 files), was collected and compared against the instrument – this comparison aimed to evaluate midwives’ record-keeping accuracy and the researcher’s CTG interpretation knowledge. This was the chosen analysis method developed by Norisus in 2003, which uses the measurement of agreement between the subject (participant) and the judge (researcher). This method was selected because the researcher is a trained midwife specialist who can judge midwives’ knowledge. The researcher maintained the worksheet’s privacy, sending it encrypted to the statistician and supervisor. No significant modifications to the worksheet or tool were required, and the supervisor and statistician approved the commencement of data collection. As the pilot study yielded no changes to the instrument, its findings were integrated into the main study results.

### Data collection

Data were collected using a checklist questionnaire to assess the records made by midwives on patients’ files. The checklist comprised two sections: demographics and CTG variables as seen in Appendix 1. Demographic data included high-risk file status, professional nurse, or midwifery specialist designation, presence of CTG in the file, clarity of notes, and delivery date (Table 1). Cardiotocography variables included baseline, beat-to-beat variability, accelerations, decelerations, and overall assessment of the trace. The files were assessed for an important element of completeness of record-keeping, ascertaining that the record-keeping is accurate and signals nurses’ knowledge of CTG interpretation. Data were kept safe in the researcher’s laptop, and codes were made for the files to maintain the anonymity of the patients and midwives. The archives department assisted in removing the randomly selected files, and they only used and knew the file number. The checklist developed was used to survey every file selected to see if there was agreement between the researcher and the midwives. A table was created to show the complete analysis of the data collected (Table 3).

**TABLE 3:** Statistics on records made.

Valid	Frequency	%	Valid %	Cumulative %
**c1mid criterion 1 Baseline: midwife**				
Reassuring: 110–160	208	56.8	56.8	56.8
Non-reassuring: 100–109	8	2.2	2.2	59.0
No record	150	41.0	41.0	100.0
**Total**	**366**	**100.0**	**100.0**	-
**c2mid Criterion 2 Variability: midwife**				
Reassuring: 5–15	3	0.8	0.8	0.8
Non-reassuring: < 5 for > 40 min but < 90 min	2	0.5	0.5	1.4
No record	361	98.6	98.6	100.0
**Total**	**366**	**100.0**	**100.0**	-
**c3mid Criterion 3 Decelerations: midwife**				
Reassuring: None	99	27.0	27.0	27.0
Non-reassuring: Early decelerations, variable decelerations with > 50% of contractions for < 3 mins	11	3.0	3.0	30.1
Abnormal: Atypical variable decelerations with > 50% of contractions, > 30 min	7	1.9	1.9	32.0
Abnormal: Late decelerations, > 30 min	3	0.8	0.8	32.8
No record	246	67.2	67.2	100.0
**Total**	**366**	**100.0**	**100.0**	-
**c4mid Criterion 4 Accelerations: midwife**				
No record	366	100.0	100.0	100.0
**c5mid Criterion 5 Assessment of trace: midwife**				
Reassuring: Norma (1 x 4)l	231	63.1	63.1	63.1
Suspicious (2 x 1)	18	4.9	4.9	68.0
Pathological (4.3 x 1)	3	0.8	0.8	68.9
No record	114	31.1	31.1	100.0
**Total**	**366**	**100.0**	**100.0**	-

*Source*: Dlamini, S., 2025, ‘Determining midwives’ knowledge of cardiotocography interpretation in selected public hospital in Gauteng: A retrospective study’, Master’s thesis, University of Johannesburg, University of Johannesburg Institutional Repository, viewed n.d., from https://hdl.handle.net/10210/516532

### Data analysis

Data analysis involved organising, reducing, and statistically testing the information obtained during data collection (Gray & Grove 2021). The researcher organised, reduced, and captured the data, then analysed it using Excel spreadsheet software. To assess the reliability of the checklist and ensure consistent interpretation across multiple reviewers, measuring agreement was essential. Drawing on the statistical principles outlined by Norušis (2003), methods such as Cohen’s Kappa or percentage agreement were used to evaluate inter-rater reliability (IRR). These methods help determine whether different coders reviewing the same CTG documentation reached similar conclusions about midwives’ recorded interpretations.

### Validity

The researcher organised, reduced, and captured the data, then analysed it using Excel spreadsheet software. To ensure consistency in observational ratings from multiple coders, an evaluation of IRR was essential (Hallgren 2012). Since the study focused on collecting data retrospectively, the checklist was modified to observe variables specific to this research. The modifications were made with the assistance of a statistician to ensure alignment with the study’s criteria. A second section was added to the checklist, transforming it into a questionnaire that the researcher completed after reviewing clinical notes and CTG tracings. A pilot study was conducted to test the clarity and relevance of the checklist, thereby supporting its validity and reliability.

### Informed consent

The researcher signed the waiver of consent form due because of the use of the patient’s records. The ethics committee accepted the waiver, and informed consent was not obtained.

### Privacy and confidentiality

The study used clinical information about women who delivered in the hospital and the clinical staff members responsible for midwifery care, whose identities must be protected. To maintain privacy and confidentiality, the researcher generated alphanumeric codes for each clinical record file, ensuring that the patients’ identities and the midwives who attended the case remained anonymous. The research information was kept in a password-encrypted document accessible to the researcher and supervisors. The researcher used statistics to report the findings, eliminating the need to name a specific clinical record, thus protecting the identity of patients and clinical staff.

### Ethical considerations

An application for full ethical approval was made to the University of Johannesburg, Faculty of Health Sciences Research Ethics Committee and ethics consent was received on 10 September 2024. The ethics approval number is REC-2988-2024. This study will adhere to the following principles: informed written consent, privacy, and confidentiality.

## Results

**FIGURE 1:**
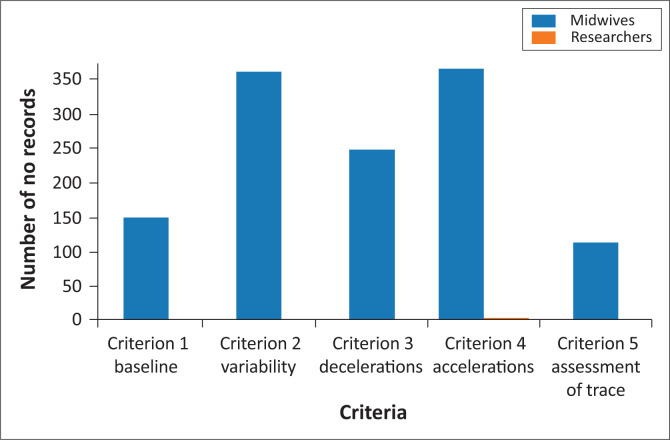
Cardiotoccography record keeping by midwives and researcher.

The CTG traces from 336 case records were analysed under five CTG criteria: baseline heart rate, variability, decelerations, accelerations, and overall trace assessment. The analysis of the CTG data reveals significant discrepancies in record-keeping between midwives and researchers. The researchers maintained complete records and interpretations of all five CTG criteria. There were noticeable recording and interpretation gaps in midwives’ documentation across CTG criteria, ranging from 31% to 100%. There are missing records in all the variables measured in the CTG interpretation of midwives as seen in Table 4 and the difference between the missing records was tabled in Figure 1 to show the lack of knowledge that leads to missing records. These gaps can compromise the midwives’ diagnosis, clinical decision-making, and neonatal outcomes. The CTG criteria used to analyse the record and interpretation of the CTG are summarised in Table 3.

**TABLE 4:** Summary of the cardiotocography criterion.

Criterion	Total number of cases out of 366	%	Rationale
Baseline	150	41.0	Missing records
Variability	361	98.6	Missing records
Decelerations	246	67.2	Missing records
Accelerations	366	100.0	Missing records
Assessment of trace	114	31.1	Missing records

### Criterion 1: Baseline

Under this criterion, midwives recorded reassuring heart rates (110 bpm – 160 bpm) in 56.8% of cases, non-reassuring rates (100 bpm – 109 bpm) in 2.2%, and had *no records for 150 cases (41.0%)*. In contrast, researchers documented reassuring heart rates in 94.0% of cases, non-reassuring rates (100 bpm – 109 bpm) in 2.5%, and non-reassuring rates (161 bpm – 180 bpm) in 3.6%, with no missing records.

### Criterion 2: Variability

Midwives recorded reassuring variability (5 bpm–15 bpm) in only 0.8% of cases, non-reassuring variability (< 5 bpm for > 40 min but < 90 min) in 0.5%, and had *no records for 361 cases (98.6%)*. Researchers documented reassuring variability in 85.0% of cases, non-reassuring variability in 13.4%, and abnormal variability (< 5 bpm for ≥ 90 min) in 1.6%, with no missing records.

### Criterion 3: Decelerations

Under this criterion, midwives recorded no decelerations in 27.0% of cases, non-reassuring early decelerations in 3.0%, abnormal atypical variable decelerations in 1.9%, abnormal late decelerations in 0.8%, and had *no records for 246 cases (67.2%)*. Researchers documented no decelerations in 85.2% of cases, non-reassuring early decelerations in 3.6%, abnormal atypical variable decelerations in 9.6%, abnormal late decelerations in 0.8%, and abnormal single prolonged decelerations in 0.8%, with no missing records.

### Criterion 4: Accelerations

The midwives had *no records for 366 cases (100.0%)*, whilst researchers recorded reassuring accelerations in 72.4% of cases, non-reassuring absence of accelerations in 27.0%, and had *no records for 2 cases (0.5%)*.

### Criterion 5: Assessment of trace

Under this criterion, midwives recorded reassuring traces in 63.1% of cases, suspicious traces in 4.9%, pathological traces in 0.8%, and had *no records for 114 cases (31.1%)*. Researchers documented reassuring traces in 72.5% of cases, suspicious traces in 13.2%, and pathological traces in 14.3%, with no missing records.

The study results demonstrate significant gaps in midwives’ recording and interpretation of the CTG. In criterion 1, the midwives’ inconsistent recording of the baseline foetal heart rate is evidenced by the disparity between their records and the researcher’s analysis. This finding suggests under-reporting of cases such as foetal bradycardia and tachycardia, which are significant in identifying the inadequacies in foetal oxygenation. Criterion 2 revealed that the record of variability was missing in 98.6% cases, suggesting that 13.4% of cases of poor variability diagnosed by the researchers were undiagnosed. The absence of deceleration records in 67.2% cases suggests that the foetal distress evidenced by late decelerations was not excluded. The absence of records and interpretations of accelerations in criterion 4 suggests a disregard for foetal physiological responses during responses to labour.

## Discussion

The study shows gaps in midwives’ recording and interpreting the CTG across all five criteria recommended by the International Federation of Gynaecology and Obstetrics for comprehensive analysis of intrapartum foetal wellbeing. This finding suggests that midwives are contravening the guidelines of intrapartum foetal monitoring and practice regulations on clinical recording. In addition, the midwives’ knowledge of the interpretation of the CTG is under scrutiny. The incomplete recording and interpretation of the CTG compromises clinical decision-making, patient safety, and the quality of record-keeping, with implications for the midwives’ professional practice.

### Compromised clinical decision-making

The study found that midwives were inconsistent in recording their interpretations of the CTG. Literature suggests that the clinical records must be written chronologically to provide a comprehensive picture to guide the health professional’s clinical decision-making (Lee et al. 2024). This finding suggests that midwives’ inconsistent recording may lead to an unclear clinical picture and misguided clinical decision-making to address the ongoing foetal compromise (Daydulo et al. 2020). Consequently, midwives may have had inappropriate responses to the cases of foetal distress, as they rely on complete records to make sound clinical decisions to guide their interventions (Nakao et al. 2020). The literature on the perinatal problem identification program names midwives’ inappropriate response to the presence of foetal distress as a personnel-related, avoidable factor (Tukisi 2023).

### Compromised patient safety

The study showed that the midwives were inconsistent in recording their interpretation of the baseline heart rate, variability, decelerations, accelerations, and overall trace assessment. The literature on CTG monitoring and interpretations suggests that the five parameters must be analysed and interpreted collectively to conclude the final diagnosis of the CTG (Chandraharan et al. 2024; Daydulo et al. 2020). In addition, Olofsson, Norén and Carlsson (2018) affirms that each criterion of the CTG is diagnostic of a specific foetal condition and has clinical significance. The baseline heart rate refers to the average foetal heart rate over 10 min, which ranges between 110 and 160 beats per minute, allowing the diagnosis of foetal tachycardia and bradycardia, which may suggest the presence of foetal distress (Zoeller & Patel 2023). Variability, on the other hand, refers to the fluctuations in the foetal heart rate, which signifies the ability of the foetal neurological system to respond to mild hypoxia induced by the uterine contractions (Crequit et al. 2022). The decelerations are the temporary and rapid drop in the foetal heart rate below the baseline, indicating a sudden cut-off in the foetal blood supply to the foetus (López-Justo et al. 2021). The accelerations refer to the temporary rapid increase in the foetal heart rate from the baseline, indicating that the foetus is not subjected to intrauterine hypoxia (Michaeli et al. 2021). López-Justo et al. (2021) affirms that baseline, variability, and decelerations are interpreted collectively to provide an overall assessment of the foetal condition. The discrepancies in the recording and interpretation of the criteria suggest that the midwives may have missed interpreting the intrauterine foetal hypoxic status (Michaeli et al. 2021). The literature suggests that intrauterine hypoxia exposes the foetus to perinatal hypoxic-ischaemic encephalopathy with detrimental effects on the foetal neurological status, which can lead to cerebral palsy (Chandraharan et al. 2024). In addition, the intrauterine foetal death and early neonatal death may result in a direct compromise of patient safety (Loussert et al. 2023).

### Quality of record-keeping

The study’s results showed that the midwives were inconsistent in recording their interpretations of the CTG monitored during the intrapartum care. The CTG trace forms part of the intrapartum records guided by the principles of record-keeping set out in the scope of practice of the registered nurse and midwives and conditions for midwives’ practice (SANC 1993, 2022; South African Government, 2022). These regulations state that midwives should keep complete, transparent, and accurate clinical records descriptive of assessments, diagnoses, treatments, and any significant changes in the patient’s condition (SANC 2022). Therefore, the midwives’ failure to consistently record their interpretations of the CTG compromises the quality of the clinical records. This finding supports the already reported outcry about the decline in the quality of clinical record-keeping in the literature (Loussert et al. 2023).

According to the results, the midwives not only compromise the quality of clinical record-keeping. However, they are also contravening their practice regulations from SANC, which could lead to their sanctions regarding acts and omissions (SANC 2014). The midwives should record their interpretations of a specific criterion, e.g. decelerations, suggesting that the midwife did not interpret clinical information that could lead to a possible intrauterine foetal hypoxia diagnosis. Consequently, midwives could be unable to draw a relevant clinical judgement to avoid conditions such as cerebral palsy and neurological injuries, which expose the midwives to litigation (Crequit et al. 2022). Therefore, midwives’ failure to record clinical data can be used as an aggravation during such litigations.

### Recommendations

The researcher elucidates gaps and inconsistencies in midwives’ recording of their interpretation of the CTG, which may suggest a knowledge deficit on the part of the midwives. It is an expectation for all five criteria to be consistently assessed, interpreted, and recorded. The study recommends that midwives be empowered in CTG interpretation and recording. This highlights the need for comprehensive and standardised training programmes to ensure that all midwives consistently understand CTG interpretation. Therefore, regular training and certification can help bridge this gap and improve the accuracy of data recording (Willis, Hrdina & Luiselli 2020). The inconsistencies in recording have negative implications for the patient’s safety because of misinterpretation, potentially resulting in inappropriate interventions or missed opportunities for timely action. The potential risks to the patients’ safety call for standardising the existing hospital protocols on foetal monitoring to ensure mandatory recording of all five criteria of the CTG. The patients’ safety concerns suggest a need for the midwives to be re-empowered on the ethical principles and considerations guiding their practice.

### Study limitations

The study evaluated midwives’ knowledge of CTG interpretation, and gaps regarding midwives’ recording were identified. However, the study was quantitative, and the midwives could not qualitatively account for the reasons for missing their interpretations of the CTG record. The study did not assess the midwives’ reactions to each CTG trace and neonatal outcome, which provided direction and indication of the midwives’ knowledge and insights into the CTG interpretations. The researchers and the midwives focused on the five criteria that directly assess the foetal wellbeing and excluded the specific monitoring, analysis, and interpretation of the uterine action with direct impact on the woman in labour. The CTG has a tocodynamometer used to monitor the uterine activity to diagnose hypotonic and hypertonic uterine contractions that could threaten the safety of the woman in labour and the foetus. The study focused on the midwives’ intrapartum recording and interpretations of the CTG. It excluded obstetricians whose clinical decision-making and plans of intrapartum care and interventions are guided by the CTG interpretations. Lastly, the study was limited to a single hospital; therefore, the findings cannot be generalised to other hospital sites.

### Strengths

The retrospective design is practical and ethical, allowing the researchers to analyse and interpret the CTG from the clinical records whilst allowing the continuity of care. The study’s findings highlight gaps in midwives’ knowledge regarding the electronic intrapartum foetal monitoring and interpretation. These gaps may be used as a point of departure towards empowerment of the midwives to render safe intrapartum care, thus reducing the deleterious maternal and neonatal outcomes. The study was limited to a single hospital, which provides deep insights relevant to the context. Consequently, the identified gaps may be used as an audit report to guide the formulation of context-specific recommendations for quality improvement in CTG interpretation and recording amongst the midwives.

## Conclusion

The analysis reveals a stark contrast in documentation completeness between midwife and researcher assessments of CTG interpretation records, with midwives showing significant gaps – most notably 98.6% missing data for variability and 100% for accelerations – whilst researcher records are nearly complete. This discrepancy raises serious concerns about the reliability of intrapartum monitoring and the potential for compromised clinical decision-making. From a researcher’s perspective, these findings highlight an urgent need for targeted training, standardised documentation protocols, and supportive systems to enhance midwives’ capacity for accurate and consistent CTG interpretation. Strengthening these practices is essential to ensure patient safety, improve perinatal outcomes, and uphold the quality of maternal care.

## Appendix 1

### Instrument part 1

#### Criteria to be measured

According to the guidelines of CTG interpretation adopted from the women’s hospital in Sweden, it is used as an instrument at KZN hospitals. The tool was modified to use the variables of the CTG as the criterion. The criteria have codes for interpretation, which will be compared to the retrospective survey carried out by the researcher. On the tool, M will represent the interpretation of the midwife/advanced midwife, R will represent the retrospective interpretation of the researcher, and NR will represent the absence of recording noted on the file.

Original tool with criteria and codes added.

**TABLE 1-A1:** Cardiotoccography Interpretation Variables.

Features (codes)	Criterion 1 Baseline BL	Criterion 2 Variability VL	Criterion 3 Decelerations DL	Criterion 4 Accelerations AL	Criterion 5 Assessment of trace AT
Reassuring	110-160 Code (1)	5-15 Code (1)	None Code (1)	Present Code (1)	Normal (1x4) Code (1)
Non-reassuring	100-109 Code (2)	< 5 for > 40 mins but < 90 min Code (2)	Early decelerations variable decelerations with > 50% of contractions for < 3mins Code (2)	No accelerations Code (2)	Suspicious (2X1) Code (2)
	161-180 Code (3)	-	-	-	-
Abnormal	< 100 > 180 sinusoidal pattern Code (4)	< 5 for > 90 mins Code (3)	Atypical variable decelerations with > 50% of contractions, > 30 mins Code (3)	-	Pathological (4.3X1) Code (3)
	-	-	Late decelerations > 30 mins Code (4)	-	-
	-	-	Single prolonged decelerations for > 3 min Code (5)	-	-
No Record	Code (5)	Code (4)	Code (6)	Code (3)	Code (4)

### Instrument part 2

#### Modified tool

**TABLE 1-A2:** Cardiotoccography Interpretation Variables.

Patients name	Patients number	File number	Criterion 1 Baseline BL		Criterion 2 Variability VL		Criterion 3 Decelerations DL		Criterion 4 Accelerations AL		Criterion 5 Assessment of trace AT	
-	-	-	Code M	Code R	Code M	Code R	Code M	Code R	Code M	Code R	Code M	Code R

## References

[CIT0001] Chandraharan, E., Pereira, S., Ghi, T., Gracia Perez-Bonfils, A., Fieni, S., Jia, Y.-J. et al., 2024, ‘International expert consensus statement on physiological interpretation of cardiotocograph (CTG): First revision (2024)’, *European Journal of Obstetrics, Gynecology, and Reproductive Biology* 302, 346–355. 10.1016/j.ejogrb.2024.09.034 39378709

[CIT0002] Crequit, S., Tataru, C., Coste, E., Diane, R., Lefebvre, M., Haddad, B. et al., 2022, ‘Association of fetal heart rate short-term variability pattern during term labor with neonatal morbidity and small for gestational age status’, *European Journal of Obstetrics & Gynecology and Reproductive Biology* 278(1), 77–89. 10.1016/j.ejogrb.2022.08.026 36126423

[CIT0003] Das, A., Majumdar, M.K. & Dey, A.K., 2023, ‘Relationship of cardiotocography and umbilical artery doppler findings with perinatal outcome in low risk pregnancies with decreased fetal movements’, *European Journal of Cardiovascular Medicine* 13(1), 702–720, viewed 29 August 2025, form https://research.ebsco.com/linkprocessor/plink?id=807530ea-6b69-3a7f-aeb2-a05f1d94af6a.

[CIT0004] David, A.L. & Spencer, R.N., 2022, ‘Clinical assessment of fetal well-being and fetal safety indicators’, *Journal of Clinical Pharmacology* 62(S1), S67–S78. 10.1002/jcph.2126 36106777 PMC9544851

[CIT0005] Daydulo, Y.D., Thamineni, B.L., Dasari, H.K. & Aboye, G.T., 2022, ‘Deep learning based fetal distress detection from time-frequency representation of cardiotocogram signal using Morse wavelet: Research study’, *BMC Medical Informatics and Decision Making* 22(1), 329. 10.1186/s12911-022-02068-1 36517791 PMC9749291

[CIT0006] Gray, J. & Grove, S.K., 2021, *Burns and Grove’s the practice of nursing research: Appraisal, synthesis, and generation of evidence*, 9th edn., Elsevier, St. Louis, MO.

[CIT0007] Hallgren, K.A., 2012, ‘Computing inter-rater reliability for observational data: An overview and tutorial’, *Tutorials in Quantitative Methods for Psychology* 8(1), 23–34. 10.20982/tqmp.08.1.p023 22833776 PMC3402032

[CIT0008] Lee, C., Kumar, S., Vogt, K.A. & Meraj, S., 2024, ‘Improving clinical documentation with AI: A comparative study of Sporo AI scribe and GPT-4o mini’.

[CIT0009] López-Justo, C., Pliego-Carrillo, A.C., Ledesma-Ramírez, C.I., Mendieta-Zerón, H., Peña-Castillo, M.Á., Echeverría, J.C. et al., 2021, ‘Differences in the asymmetry of beat-to-beat fetal heart rate accelerations and decelerations at preterm and term active labor’, *Sensors* 21(24), 8249. 10.3390/s21248249 34960343 PMC8704786

[CIT0010] Loussert, L., Berveiller, P., Magadoux, A., Allouche, M., Vayssiere, C. & Garabedian, C., et al., 2023, ‘Cardiotocography interpretation and clinical decision-making: Current practices and challenges in obstetrics’, *Journal of Gynecology Obstetrics and Human Reproduction* 52(10), 102622. 10.1016/j.jogoh.2023.102622 37321399

[CIT0011] Michaeli, J., Srebnik, N., Zilberstein, Z., Rotem, R., Bin-Nun, A. & Grisaru-Granovsky, S., 2021, ‘Intrapartum fetal monitoring and perinatal risk factors of neonatal hypoxic–ischemic encephalopathy’, *Archives of Gynecology & Obstetrics* 303(2), 409–417. 10.1007/s00404-020-05757-2 32870345

[CIT0012] Nakao, M., Okumura, A., Hasegawa, J., Toyokawa, S., Ichizuka, K., Kanayama, N. et al., 2020, ‘Fetal heart rate pattern in term or near-term cerebral palsy: A nationwide cohort study’, *American Journal of Obstetrics & Gynecology* 223(6), 907.e1–907.e13. 10.1016/j.ajog.2020.05.059 32497609

[CIT0013] Norušis, M.J., 2003, *SPSS 12.0 guide to data analysis*, Prentice Hall, Upper Saddle River, NJ.

[CIT0014] Olofsson, P., Norén, H. & Carlsson, A., 2018, ‘New FIGO and Swedish intrapartum cardiotocography classification systems incorporated in the fetal ECG ST analysis (STAN) interpretation algorithm: Agreements and discrepancies in cardiotocography classification and evaluation of significant ST events’, *Acta Obstetricia et Gynecologica Scandinavica* 97(2), 219–228. 10.1111/aogs.13277 29215160 PMC5887886

[CIT0015] Rishard, M., Weerasundara, I., Fonseka, R., De Abrew, A., Wijesinghe, M.S.D., Senanayake, H. et al., 2025, ‘Development and evaluation of an online cardiotocography course tailored to LMIC settings: A feasibility study conducted in a tertiary care hospital in Sri Lanka’, *BMC Research Notes* 18(1), 1–7. 10.1186/s13104-025-07239-7 40221771 PMC11993943

[CIT0016] Rosen, H. & Yogev, Y., 2023, ‘Assessment of uterine contractions in labor and delivery’, *American Journal of Obstetrics & Gynecology* 228(5), S1209–S1221. 10.1016/j.ajog.2022.09.003 37164494

[CIT0017] Sellers, P.M., Dippenaar, J. & Da Serra, D., 2018, *Sellers’ midwifery*, Juta & Company Ltd, Cape Town, viewed 29 August 2025, from https://research.ebsco.com/linkprocessor/plink?id=1ce98ad1-7653-379d-8032-33641624ab65.

[CIT0018] South African Government, 2022, *Nursing Act No. 33 of 2005, Government Notice 2127, Regulations regarding the scope of practice for nurses and midwives, Government Gazette No. 46471*, p. 14, Government Printing Works, Pretoria.

[CIT0019] South African Nursing Council (SANC), 1993, *Regulations relating to the conditions under which registered midwives and enrolled midwives may carry on their profession (Government Notice No. R.2488 of 26 October 1990)*, Government Printer, Pretoria, viewed 27 June 2025, from https://www.sanc.co.za/r-2488/ [R. 2488 – SANC].

[CIT0020] South African Nursing Council (SANC), 2014, *Regulations relating to the acts and omissions for registered nurses and midwives, Government Gazette, No. 38047, (Government Notice R. 767, 01 October 2014)* viewed from https://www.sanc.co.za/wp-content/uploads/2020/06/R767-Reg-act-2014.pdf.

[CIT0021] South African Nursing Council (SANC), 2022, *Regulations relating to the conditions under which registered midwives and enrolled midwives may carry on their profession (Government Notice No. R.2488, 26 October 2022)*, Government Printer, Pretoria.

[CIT0022] The South African Department of Health’s (DoH), 2024, *National Integrated Maternal and Perinatal Care Guidelines*, 5th edn, Pretoria.

[CIT0023] Tukisi, K.P., 2023, ‘Midwives’ descriptions of avoidable causes of negative perinatal outcomes’, *Health SA Gesondheid* 28, a2185. 10.4102/hsag.v28i0.2185 PMC10713483 38090474

[CIT0024] Willis, K., Hrdina, J. & Luiselli, J.K., 2020, ‘Performance management and maintenance of data recording by educational care providers’, *Behavior Analysis: Research and Practice* 20(3), 165–173. 10.1037/bar0000177

[CIT0025] Zoeller, B.B. & Patel, S., 2023, ‘Fetal arrhythmia: Bradycardia and tachycardia’, in R. Abdulla, J.K. Singh & A.M. Gewitz (eds.), *Pediatric cardiology*, pp. 1123–1138, Springer, Cham.

